# The Effects of Sensory Threshold Somatosensory Electrical Stimulation on Users With Different MI-BCI Performance

**DOI:** 10.3389/fnins.2022.909434

**Published:** 2022-06-17

**Authors:** Long Chen, Lei Zhang, Zhongpeng Wang, Bin Gu, Xin Zhang, Dong Ming

**Affiliations:** ^1^Department of Biomedical Engineering, Academy of Medical Engineering and Translational Medicine, Tianjin University, Tianjin, China; ^2^Department of Biomedical Engineering, College of Precision Instruments & Optoelectronics Engineering, Tianjin University, Tianjin, China

**Keywords:** motor imagery, brain-computer interface, sensory threshold somatosensory electrical stimulation, functional connectivity, EEG

## Abstract

Motor imagery-based brain-computer interface (MI-BCI) has been largely studied to improve motor learning and promote motor recovery. However, the difficulty in performing MI limits the widespread application of MI-BCI. It has been suggested that the usage of sensory threshold somatosensory electrical stimulation (st-SES) is a promising way to guide participants on MI tasks, but it is still unclear whether st-SES is effective for all users. In the present study, we aimed to examine the effects of st-SES on the MI-BCI performance in two BCI groups (High Performers and Low Performers). Twenty healthy participants were recruited to perform MI and resting tasks with EEG recordings. These tasks were modulated with or without st-SES. We demonstrated that st-SES improved the performance of MI-BCI in the Low Performers, but led to a decrease in the accuracy of MI-BCI in the High Performers. Furthermore, for the Low Performers, the combination of st-SES and MI resulted in significantly greater event-related desynchronization (ERD) and sample entropy of sensorimotor rhythm than MI alone. However, the ERD and sample entropy values of MI did not change significantly during the st-SES intervention in the High Performers. Moreover, we found that st-SES had an effect on the functional connectivity of the fronto-parietal network in the alpha band of Low Performers and the beta band of High Performers, respectively. Our results demonstrated that somatosensory input based on st-SES was only beneficial for sensorimotor cortical activation and MI-BCI performance in the Low Performers, but not in the High Performers. These findings help to optimize guidance strategies to adapt to different categories of users in the practical application of MI-BCI.

## Introduction

Brain-computer interface (BCI) constructs a communication pathway and control channel between brain activity and various devices, which enables users to interact with the external environment without relying on the muscle tissues (Wolpaw et al., 2002; Wang et al., 2019). In particular, BCIs based on electroencephalography (EEG) signals have been developed for different tasks and applications. The popular EEG-based BCI paradigms include steady state visual evoked potential (SSVEP), P300 event-related potential (ERP), motor imagery (MI), etc (Wang et al., 2019). As a mental rehearsal of limb movement, MI can induce neural activations over sensorimotor regions. The cortical activities generated by MI are usually observed as event-related desynchronization (ERD) or synchronization (ERS) in alpha and beta rhythms that can be detected and used for BCI control (Pfurtscheller and Neuper, 2001; Jeon et al., 2011). The BCIs based on MI (MI-BCIs) promote motor-related cortical plasticity and have been widely used in the field of motor rehabilitation and motor learning. Several studies have shown that MI-BCIs are effective in functional improvements of limb and drive significant recovery in stroke patients (Mrachacz-Kersting et al., 2016; Biasiucci et al., 2018).

Although MI-BCIs have a promising prospect in stroke rehabilitation, many users seem unable to produce ideal brain activity for the BCI control. There is about 15–30 percent of subjects are incapable of controlling a BCI at all, this lack of control is termed as “BCI illiteracy” or “BCI inefficiency” phenomenon (Allison and Neuper, 2010). Meanwhile, the MI-BCI performance of the most of remaining BCI literate participants was also mediocre (Jeunet et al., 2014). Therefore, various strategies need to be developed to improve MI-BCI performance and facilitate the practical applications of MI-BCI. In recent years, many researchers have devoted themselves to developing advanced machine learning algorithms to increase the decoding accuracy of brain signals. As invented for binary classification problems, Support Vector Machine (SVM) is capable of separating EEG signals between two classes by building a hyperplane with the largest margin (Bhuvaneswari and Kumar, 2013). In addition to traditional algorithms, deep learning methods are also introduced in MI-BCI to increase the classification accuracy (Zhao et al., 2019). Although, these advanced algorithms have achieved a slew of promising results in MI patterns recognition applications, the achievement of higher decoding accuracy is still limited by the inability of some subjects to produce reliable EEG responses (Ren et al., 2020; Li et al., 2021). This may be due to the subjects' inability to perform MI tasks properly. Therefore, several research works focused on developing appropriate MI guidance strategies to assist subjects to perform MI efficiently and accurately. Choi et al. found that providing a virtual reality (VR) as guidance induces neural patterns with greater discriminability (Choi et al., 2020). Similar results were also reported by Škola et al. where VR-based visual guidance succeeded in the improvement of the MI-BCI performance (Škola et al., 2019). In addition, somatosensory afference, which is essential for building internal body representation in MI tasks, has also attracted much attention recently. Compared with visual guidance, somatosensory afference is a more natural guidance strategy for subjects (Cincotti et al., 2007). Yao et al. demonstrated that enhancement of MI-BCI performance can be achieved by using tactile stimulation to optimize guidance strategy (Yao et al., 2013). Shu et al. showed in stroke patients that the application of tactile stimulation to the ipsilateral wrist achieved stronger motor-related cortical activation (Shu et al., 2018a). As a promising tool for motor rehabilitation, neuromuscular electrical stimulation (NMES) can induce muscular contraction and convey somatosensory afference. Yi et al. demonstrated that electrical stimulation combined with MI can increase the decoding accuracy of neural patterns (Yi et al., 2017). Reynolds et al. showed that the combination of MI and NMES induces a stronger sensorimotor rhythms ERD than MI alone (Reynolds et al., 2015). Although somatosensory stimulation is appropriate to improve motor imagery patterns, stimulation with excessive intensity may interfere with MI patterns and makes users unable to concentrate on imagining movement (Corbet et al., 2018). Recently, Tu-Chan et al. showed that sensory threshold somatosensory electrical stimulation (st-SES) can modulate activity in the sensorimotor cortices, and thus promote the recovery of hand motor function (Tu-Chan et al., 2017). NMES involves the application of repetitive transcutaneous electrical stimulation to superficial skeletal muscles, with the main objective to generate visible muscle contractions by depolarizing motor axons. In the same way that motor axons are recruited by NMES, sensory axons are also depolarized (Bergquist et al., 2011). However, devices such as NMES units can also deliver sensory threshold stimulation. Compared with conventional NEMS, st-SES can convey proprioceptive signals primarily by activating sensory axons without triggering large muscular contraction. Veldman et al. observed that a larger activation of sensorimotor regions and cortical connectivity was associated to somatosensory inputs in the form of st-SES (Veldman et al., 2018). Corbet et al. showed that st-SES during MI increased connectivity between the frontal-parietal network and significantly improved the accuracy of classifier for discriminating MI from resting state (Corbet et al., 2018). These studies demonstrated that st-SES is an effective way to modulate the brain patterns of somatosensory cortices and promote BCI performance.

Although past studies have shown that the guidance based on somatosensory afference contributes to the improvement of BCI performance, somatosensory afference guidance does not appear to be effective for all subjects. Park et al. reported that BCI illiterate subjects achieve significantly higher MI-BCI classification accuracy when subjects are asked to perform the somatosensory-motor imagery, but BCI literate subjects experience a slight decrease in classification performance (Park et al., 2021). In addition, Kaiser et al. showed that cortical effects of BCI training are only found in BCI illiterate subjects but not in BCI literate subjects (Kaiser et al., 2011). This means that some guidance strategies may not be suitable for all users. However, most of the previous studies have not explored the impact of somatosensory afference guidance on different categories of subjects. To our knowledge, no study has reported the different effects of somatosensory stimulation on MI performance in high and low BCI performers, which is not conducive to adapt the guidance strategy to each user. Therefore, although st-SES has shown great potential in fostering BCI performance, it is worth exploring whether st-SES is appropriate to assist subjects with different BCI performance.

In this study, we aim to investigate the effect of combining MI and st-SES on cortical activation and MI-BCI classification performances in high and low BCI performers. Considering the physical and psychological differences between high and low BCI performers (Shu et al., 2018b), we presume that st-SES have different effects on the two categories of subjects. In addition, Corbet et al. demonstrated the st-SES is feasible in improving the accuracy of discriminating MI combined with st-SES and resting state (Corbet et al., 2018). However, the stimulation effect of st-SES on a brain-switch BCI used to distinguish between task state and resting state is still unclear. Thus, the current study focused on evaluating brain-switch BCI performance when st-SES was constantly applied during both MI task and resting state. Offline BCI performances were evaluated with or without st-SES modulation. Further, to investigate the effect of st-SES on cortical activation, we compared the activation intensity of the sensorimotor cortex between MI combined with st-SES and MI alone.

## Materials and Methods

### Participants

Twenty able-bodied subjects (11 males, mean age 23.4 ± 0.6, right-handed) participated in this study. All participants have no history of neurological or psychiatric disorders. The experimental procedure described in this study was approved by the ethical committee of Tianjin University. The detailed process of the experiment was clearly explained to each participant before experimental data were recorded. All participants signed an informed consent form.

### EEG Recording and St-SES Intervention

During the whole experiment, subjects were instructed to sit in a relaxed position about 1.5 m in front of the screen with palms facing up. EEG signals were recorded using the SynAmps2 system (Neuroscan, Victoria, Australia) with 60 standard Ag/AgCl electrodes, which were placed on the scalp according to the international 10–20 system. A ground electrode was placed on the forehead and a reference electrode was placed on the nose. EEG signals were recorded at a sampling rate of 1,000 Hz and a notch filter with 50 Hz was used in the acquisition process. The impedances were kept under 10 kΩ during the data acquisition.

St-SES was generated using VitalStim Therapy (Chattanooga Group, TN, USA). Two electrodes were placed on the participants' flexor digitorum superficialis at the anterior face of the right forearm. The frequency of stimulation was set to 30 Hz for all participants. The stimulation amplitude of st-SES was individually evaluated for each participant before EEG recordings. The amplitude was gradually increased from 0 mA until the participants reported a closure sensation of the affected hand, while the amplitude was adjusted to avoid eliciting any visible movement. The stimulation intensity varied between 4 and 6 mA according to the subject-dependent sensory threshold.

### Design of the Experimental Paradigm

This study contained four different experiment conditions (MI, SES, MI-SES, and Rest). Before the experiment began, participants performed gripping movements to familiarize themselves with the imaginary proprioceptive sensations. For MI and MI-SES conditions, the instructions given to the participants were the following: “You have to perform kinesthetic imagery of right-hand grasp movement (RH-MI) while seeing the visual guidance on the screen. During the task period, you should imagine the proprioceptive sensation of the hand closing without eliciting any muscular contraction. You should perform continuous MI instead of repetitive MI and keep a consistent MI strategy across trials.” For SES and Rest conditions, the instructions were the following: “You should keep fully relaxed and avoid any movement of limbs. You have to avoid paying attention to your hands and should not think about your hands.” In SES condition, the participants received st-SES and were instructed to remain at rest. In the MI-SES condition, the participants were instructed to perform RH-MI and st-SES was applied on the participants' forearms during MI. In MI and Rest conditions, even if the st-SES was not applied, the electrodes were anyway placed on the skin to ensure consistent tactile stimulation.

**Figure 1:**
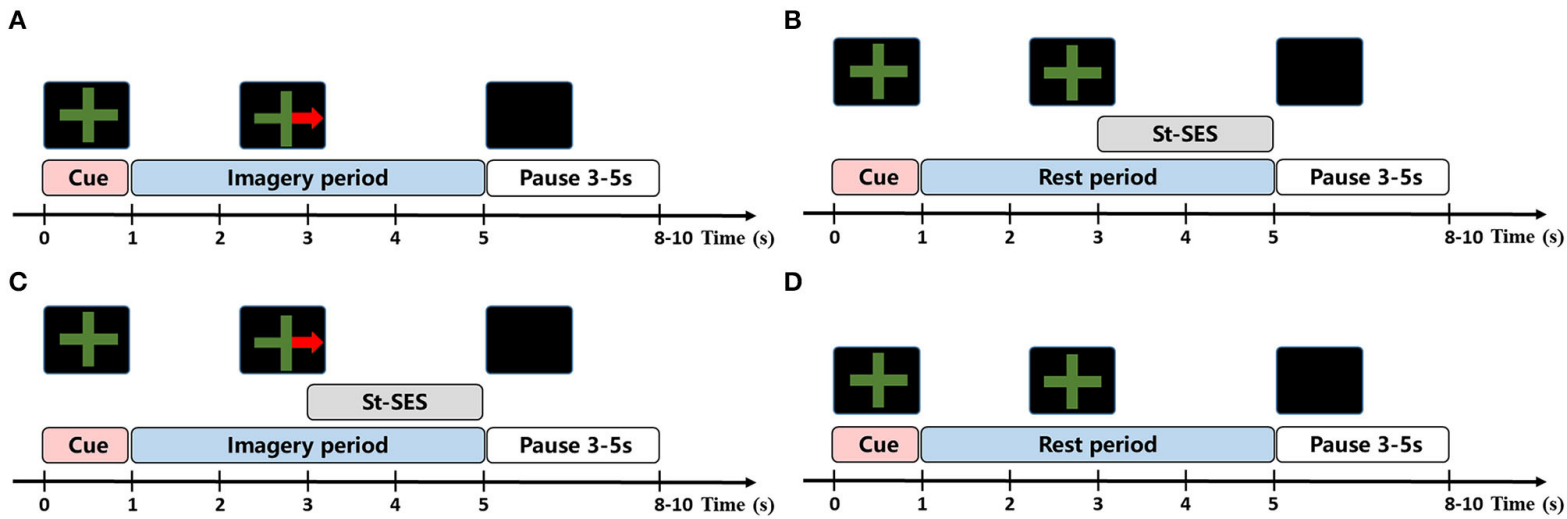
The task procedure of experimental condition. **(A)** MI condition. In each trial of MI condition, the subjects were required to perform MI task according to the cue. **(B)** SES condition. In each trial of SES condition, the subjects were asked to remain at rest and received st-SES intervention. **(C)** MI-SES condition. In each trial of MI-SES condition, the subjects were instructed to perform MI task according to the cue and st-SES was applied to subjects during MI. **(D)** Rest condition. In each trial of Rest condition, the subjects were asked to remain relax.

The experiment included four runs. Each run consisted of four conditions (10 trials per condition) and trials were performed in random order, with 40 trials per condition at the end of the experiment. The number of trials for each condition was consistent with Corbet et al.'s study to eliminate discrepancies in results that might be caused by trial size (Corbet et al., 2018). The experimental paradigm of each trial is represented in Figure 1. The trial began with a green cross which appeared on the center of the screen as a preparation cue for the task and lasted for 1 s. Then, an initiation cue in the form of a green cross with or without a red arrow is presented in the middle of the screen for 4 s, indicating the onset of the task period. During this period, participants should perform RH-MI (red arrow on the green cross) or stay at rest (green cross alone) until the cue disappeared. In order to prevent the attention on MI from being disturbed by st-SES in MI-SES condition, st-SES was not activated until 2 s after the task period onset. Similarly, in the SES condition, st-SES was activated 2 s after the task began. When the initiation cue disappeared, the screen turned black. The time interval between trials varied randomly between 3 and 5 s to prevent adaptation. The participants were advised to restrict movements such as blinking or swallowing during the task period which may produce artifacts.

### Pre-Processing

For decoding analysis, EEG signals were only filtered into 8–28 Hz and down sampled to 250 Hz. For the rest of the analysis, raw EEG data were firstly band-pass filtered in the frequency band between 0.5 and 100 Hz to remove high-frequency noise and baseline drift. The signals were then down sampled to 250 Hz. Independent component analysis (ICA) was applied to filter out the components related to ocular and electromyographic artifacts. The rank-deficiency problem was accounted for by reducing the number of ICs. We then performed the surface Laplacian transform to eliminate the low-frequency coupling among electrodes. After that, the noisy trials, which were detected post-experiment by visual inspection, were discarded in the analysis.

### Classification Analysis

EEG features of each condition were extracted by the common spatial pattern (CSP) algorithm. The CSP algorithm is commonly used to extract discriminative spatial features from EEG by maximizing the variance difference between two classes. The data of the period from 1 to 5 s after the preparation cue appeared were utilized for decoding analysis. The features were extracted from the frequency band between 8 and 28 Hz which was associated with sensorimotor rhythm. The log-variance of the first and last four components generated by CSP filters were selected to construct feature vectors. After that, a SVM classifier with a linear kernel was used to classify the users' neural patterns, with the regularization parameter C of the classifier set to the default value of 1. One classifier was trained to discriminate MI-SES condition from SES condition, and another classifier was used to discriminate MI condition from Rest condition. In addition, we also investigated the accuracy of each of the two class classifiers that discriminates MI-SES or SES conditions from the Rest condition. All classifiers were evaluated through the leave-one-out cross-validation method. The 80 trials of two class were randomly divided into forty sets. Each set included one sample of each class. In each fold, one set was selected as the testing set and the remaining samples as the training set. The final classification accuracy was the average of all fold classifications.

The participants were assigned to Low and High performers based on their classification accuracy in discriminating MI condition from Rest condition described in the previous paragraph. In this study, High performers were defined as those with a classification accuracy of more than 80%. According to the decoding accuracy, 10 participants were assigned to High performers group, and the other 10 participants were assigned to the Low performers group. Previous studies typically set the inefficiency threshold for two-class MI-based BCI (distinguishing two brain states) at 70% accuracy (Brunner et al., 2010; Shu et al., 2018b). However, Allison and Neuper showed that the inefficiency threshold should be determined based on the type of BCI (Allison and Neuper, 2010). Compared with two-class BCI, brain-switch BCI is designed to detect only one brain state (Pfurtscheller et al., 2010). In addition, some studies suggested that a threshold of at least 80% should be used to determine BCI literate subjects (Kaplan et al., 2016; Horowitz et al., 2021). Thus, we used a threshold of 80% in the brain-switch BCI.

### Time–Frequency Analysis

In order to evaluate the effect of somatosensory input on cerebral cortex activity, we computed event-related spectral perturbation (ERSP) for three conditions (MI, SES, and MI-SES). ERSP describes the power changes of EEG signals in the time-frequency domain (Makeig et al., 2004). The increase or decrease of power relative to baseline in a specific frequency band can be represented in the form of ERS or ERD. Some studies have demonstrated that somatosensory input has a significant effect on cortical activation of MI. In this study, we mainly compared the differences in ERSP values between MI and MI-SES conditions for each group. The calculation formula of ERSP for *n* trials was defined as follow:


(1) $$\begin{array}{r} ERSP\left(f,t\right)\;=\;\frac{1}{n}\overset{n}{\underset{k=1}{\sum }}F_{k}{\left(f,t\right)}^{2} \end{array}$$

where $F_{k}{\left(f,t\right)}^{\;}$ represents the spectral estimation of *k*th trial at frequency *f* and time *t*. The short-time Fourier transform (STFT) with a Hanning-tapered window from EEGlab was applied to compute the ERSP (dB) (Delorme and Makeig, 2004). Window width was set as 256 sampling points (Yi et al., 2017). The ERSP values were normalized by subtracting the mean power changes during a baseline period of [−1 −0.2] s. The average ERSP values across all subjects at key channel C3 were mainly analyzed. For quantifying the cortex activation in each condition, ERSP values at channel C3 were averaged over task time (3–5 s) and within alpha (8–13 Hz) and beta (14–28 Hz) rhythm bands. A paired *t*-test be used to compare the averaged ERSP values between MI-SES and MI conditions in each rhythm band for each group only if the normality test (Shapiro–Wilk test) was satisfied. If the normality was not satisfied, the Wilcoxon signed-rank test was used.

Further, the coefficient of determination *r*^2^ was calculated to assess the differences in signal spectra between MI and resting state with or without st-SES modulation. The *r*^2^ value is between 0 and 1, where *r*^2^ value close to 1 indicates that two states are well discriminative, and *r*^2^ value close to 0 indicates that two states can be difficult to distinguish (Meng et al., 2018). For each subject, *r*^2^ value of each channel was averaged over the frequency band of [8 28] Hz and time interval of [3 5] s, and then averaged across all subjects.

### Signal Complexity Analysis

The brain is a complex non-linear system, and neural activity measured by EEG signals exhibits non-linear dynamic properties. To estimate the complexity of the EEG signal, the sample entropy of EEG data for MI-SES and MI conditions was calculated. Sample entropy (SampEn) is the negative natural logarithm of the conditional probability that two data sequences are similar for length *m* remain similar for length *m* + 1, where a larger value corresponds to the greater probability of generating new patterns in time series and the higher complexity in sequences (Richman and Moorman, 2000). Therefore, SampEn can be used to assess the complexity of EEG during motor imagery. The SampEn was computed as follow:

For a time series *X* with *N* data points, *m* consecutive *X* data points were extracted from that to form a sequence *X*_*m*_(*i* ).


(2) $$\begin{array}{r} X_{m}\left(i\right)\;=\;\left\{x_{i},\ldots ,x_{i\;+\;m\;-\;1}\right\},\;1\leq i\leq N\;-\;m \end{array}$$

where *m* is the embedding dimension, *i* represents starting at the *i*th point. The distance between two sequence *X*_*m*_(*i*) and *X*_*m*_(*j*) is denoted as *d*[_*X*_*m*_(*i*), *Xm*_(*j*)] which is defined to be the maximum absolute difference between their corresponding scalar components. The number of all sequence pairs with the *d*[_*X*_*m*_(*i*), *Xm*_(*j*)] ≤ *r* is counted and represented as $B_{i}^{m}\left(r\right)$. The *r* denotes the tolerance and is defined as *r* = *g* × *SD*, where *SD* is the standard deviation of the time series *X*. Then, a parameter $B_{m}^{\;}\left(r\right)$ can be defined as:


(3) $$\begin{array}{r} B_{m}\left(r\right)\;=\;\frac{1}{N\;-\;m}\overset{N-m}{\underset{i\;=\;1}{\sum }}\frac{B_{i}^{m}\left(r\right)}{N\;-\;m\;-\;1} \end{array}$$

After the embedding dimension is set to *m*+1, the above process is repeated to obtain *B*_*m*+1_(*r*). Finally, the SampEn of time series is defined as:


(4) $$\begin{array}{r} SampEn\left(x,m,r\right)\;=\;-\;\ln\frac{B_{m\;+\;1}\left(r\right)}{B_{m}\left(r\right)} \end{array}$$

Here, the embedding dimension was set as *m* = 2 and the tolerance was set as *r* = 0.1 × *SD*. The baseline SampEn of each trial was computed using EEG data in [−1, −0.2] s. To obtain the relative SampEn, SampEn values within task period [3 5] s were normalized by subtracting the baseline SampEn value and divided by this same baseline SampEn value. Then the relative SampEn values of the trials in each condition were averaged. The relative SampEn values at channel C3 and within [8 28] Hz were calculated for further analysis. Then, a statistical analysis was performed to compare relative SampEn values between MI-SES and MI conditions in each group.

### Functional Connectivity Analysis

In order to inspect differences in brain area interactions between High performers and Low performers, the weighted phase lag index (wPLI) was used for functional connectivity analysis. The wPLI is a method to estimate the asymmetry in the distribution of instantaneous phase differences between two time series (Vinck et al., 2011). The larger the wPLI value, the greater the phase consistency between brain regions. The wPLI has been shown to be insensitive to zero-lag phase relations typically caused by contaminations from volume conduction. The wPLI is defined as:


(5) $$\begin{array}{r} {wPLI}_{xyt}\;=\;\frac{n^{-1}\overset{n}{\underset{t\;=\;1}{\sum }}|imag\left(s_{xyt}\right)|sign\left(imag\left(s_{xyt}\right)\right)}{n^{\;-\;1}\overset{n}{\underset{t=\;1}{\sum }}|imag\left(s_{xyt}\right)|} \end{array}$$

whereby *imag* (*s*_*xyt*_) indicates the imaginary component of the cross-spectrum between time series *x* and *y* at time point *t*, and *sign* denotes the sign function (−1, + 1 or 0).

In this case, the EEG of 30 channels overlying the frontal, sensorimotor, and parietal areas was selected to construct a connectivity network. These channels include F line channels (F1–F6), FC line channels (FC1–FC6), C line channels (C1–C6), CP line channels (CP1–CP6) and P line channels (P1–P6). Here, wPLI values were calculated from EEG data within time period of [3 5] s and frequency range of alpha (8–13 Hz) and beta (14–28 Hz) by function ft_connectivity_wpli.m implemented in the Fieldtrip toolbox (Oostenveld et al., 2011). After that, the wPLI values in each pair of channels were normalized by subtracting the mean wPLI value of baseline period [−1, −0.2] s and divided by the standard deviation of wPLI value during the baseline period. Then, the statistical significance of non-zero wPLI values was assessed by a permutation test based on the t-statistic. The significant functional connectivity between channels was defined as the *p*-value below the critical threshold of 0.005 according to previous studies (Jin et al., 2017; Li et al., 2020).

## Results

### Classification Performance

**Figure 2:**
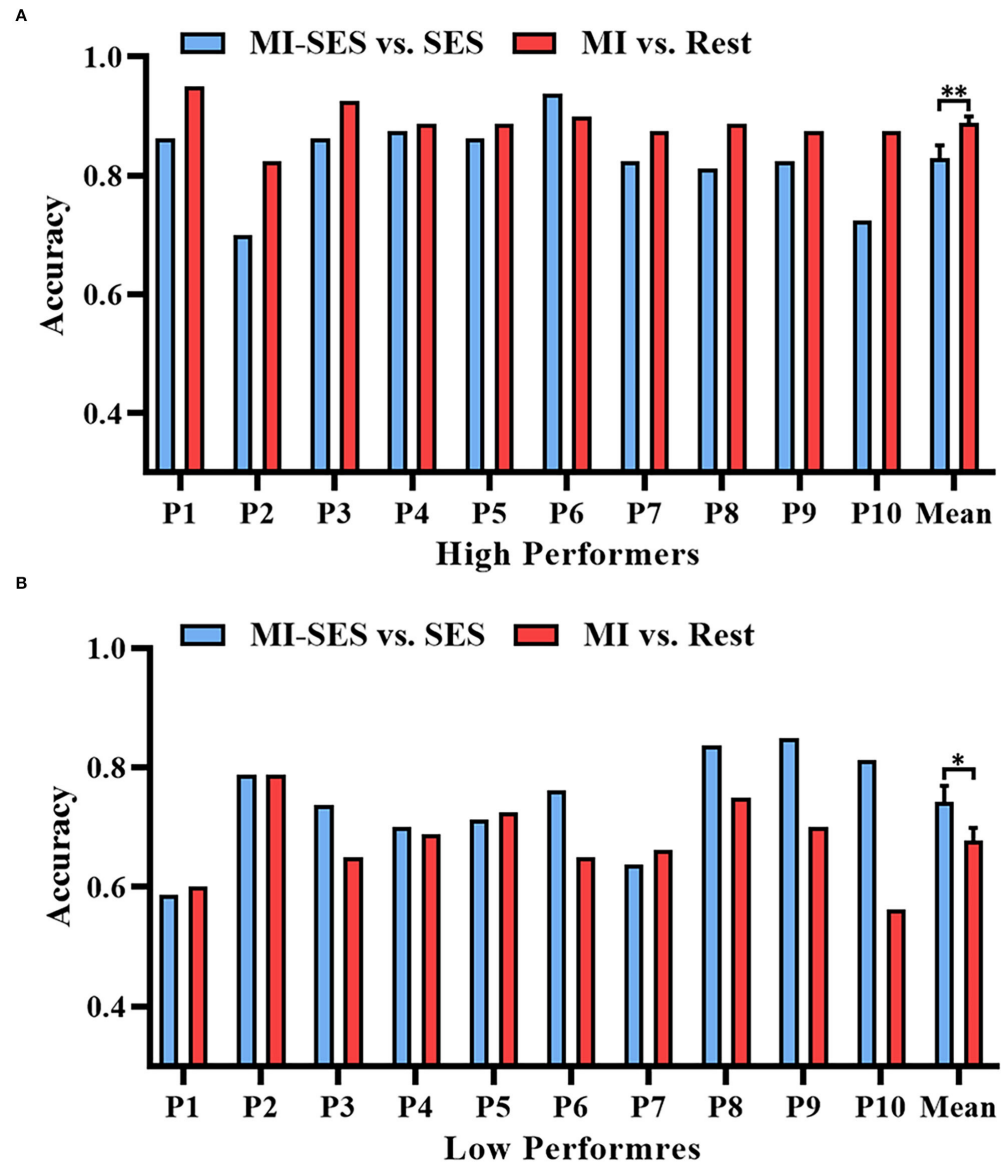
Offline accuracies of brain-switch BCI with or without st-SES modulated. **(A)** Classification accuracy results in High Performers group. **(B)** Classification accuracy results in Low Performers group. Error bars represent standard error of mean. “*” indicates *p* < 0.05, “**” indicates *p* < 0.01.

The offline accuracies of brain-switch BCI in High Performers group and Low Performers group are presented in Figure 2. The classification accuracies for both High and Low Performers groups were normally distributed, as assessed by the Shapiro–Wilk test (*p* > 0.05). We compared the performance of the MI-SES vs. SES classifier and MI vs. Rest classifier. For High Performers group, a paired *t*-test revealed a significant decrement in classification accuracy for MI-SES vs. SES classifier compared with MI vs. Rest classifier (0.83±0.07 vs. 0.89±0.03, *p* < 0.01). More specifically, nine out of 10 participants had lower classification accuracy with the st-SES intervention. However, the accuracy of MI-SES vs. SES classifier was significantly higher than that of MI vs. Rest classifier in Low Performers group (0.74 ± 0.09 vs. 0.68 ± 0.07, *p* = 0.047). Most of the Low Performers achieved better classification accuracy with the st-SES intervention, but still, three out of 10 participants showed a slight reduction in accuracy of MI-SES vs. SES classifier compared with MI vs. Rest classifier.

**Figure 3:**
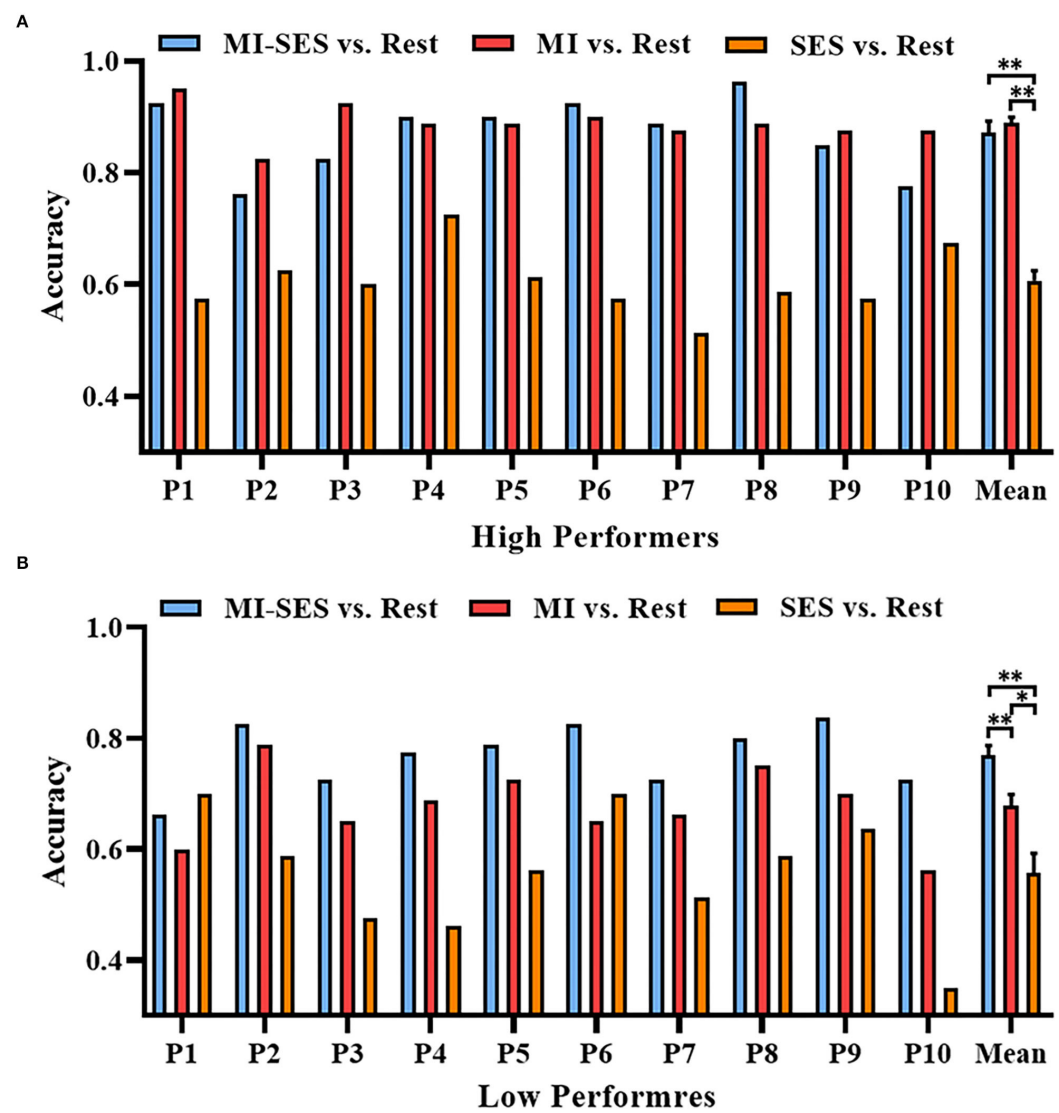
Offline accuracies of distinguishing three conditions (MI-SES, MI, SES) from Rest condition. **(A)** Classification accuracy results in High Performers group. **(B)** Classification accuracy results in Low Performers group. Error bars represent standard error of mean. “*” indicates *p* < 0.05, “**” indicates *p* < 0.01.

Furthermore, we investigated whether somatosensory input could make the neural pattern in the MI task more distinguishable from that in the resting state. We compared the classification accuracies of classifiers that distinguish three conditions (MI-SES, MI, SES) from the Rest condition in each group (Figure 3). Through one-way repeated measures ANOVA, we found that there was a significant difference in accuracy among the three classifiers in both High Performers (*F*(2, 18) = 80.3, *p* < 0.01) and Low Performers (*F*(1.3, 11.67) = 24.78, *p* < 0.01). In the High Performers group, the Bonferroni *post-hoc* analysis indicated that the accuracy of both MI-SES vs. Rest and MI vs. Rest classifiers was significantly higher than that of the SES vs. Rest classifier (0.61 ± 0.06, *p* < 0.01 both comparisons). However, there was no significant difference between MI-SES vs. Rest classifier and MI vs. Rest classifier (0.87 ± 0.07 vs. 0.89±0.03). Specifically, only five out of 10 participants achieved higher accuracy in MI-SES vs. Rest classifier as compared with the performance in MI vs. Rest classifier. For the Low Performers group, the Bonferroni *post-hoc* analysis demonstrated that MI-SES vs. Rest classifier achieved significantly higher accuracy than MI vs. Rest classifier (0.77 ± 0.06 vs. 0.68 ± 0.07, *p* < 0.01). All participants in Low Performers group achieved better classification performance in MI-SES vs. Rest classifier than in MI vs. Rest classifier. In addition, the classification performance of both MI-SES vs. Rest and MI vs. Rest classifiers was greater than that of the SES vs. Rest classifier (0.56 ± 0.11, *p* < 0.01 and *p* = 0.025, respectively). From the results of these analyses, the st-SES only improved the classification performance of the Low Performers group, but not the classification accuracy of the High Performers group.

### Time-Frequency Results

**Figure 4:**
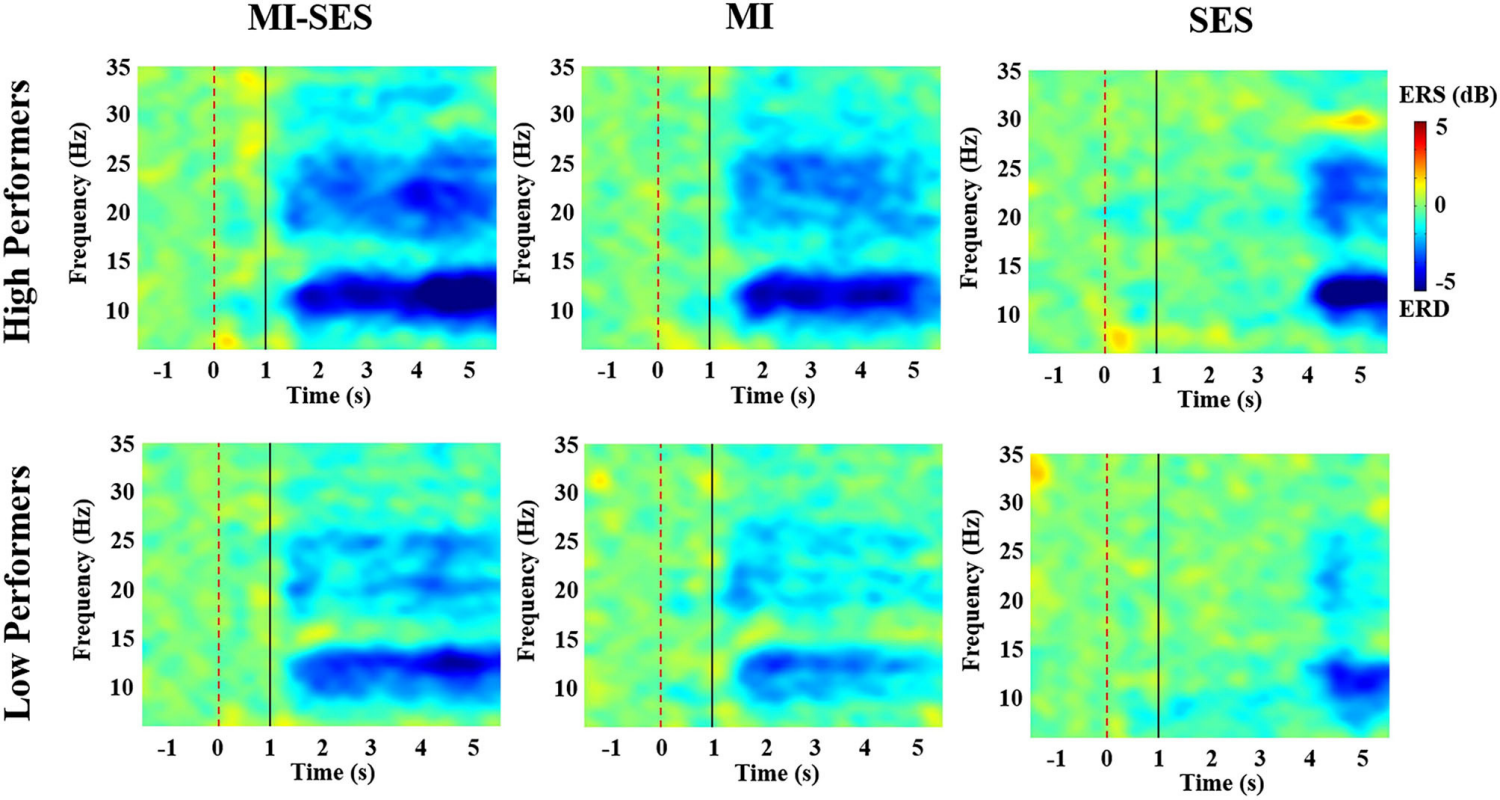
The average time-frequency maps over C3 channel for all High Performers and all Low Performers. The period [1 5] s indicated the MI or rest task. The period [3 5] s corresponds to st-SES in MI-SES and SES conditions.

In order to better understand the effect of somatosensory stimulation on neural response during MI, we analyzed the band power changes of EEG in the sensorimotor cortex under three conditions (MI-SES, MI, SES). The average time-frequency maps of ERSP values at channel C3 (in the contralateral hemisphere) in each group are presented in Figure 4. The ERSP within alpha and beta rhythms can be used as a measure of cortical activation. As shown in Figure 4, ERD could be observed in alpha and beta frequency bands for all conditions in both groups, indicating that the sensorimotor cortex was activated in all three conditions. However, more visible ERD was found in MI-SES condition after st-SES was activated compared with MI and SES conditions. This result is consistent with previous studies showing that the motor cortex excitability can be improved by the combination of MI and somatosensory stimulation (Corbet et al., 2018; Vidaurre et al., 2019). In addition, the ERD patterns during MI tasks indicated that motor cortex activation in the High Performers group was stronger than that in the Low Performers group. Moreover, compared with High Performers group, the Low Performers group had difficulty maintaining a consistent ERD pattern during the task period under MI conditions.

**Figure 5:**
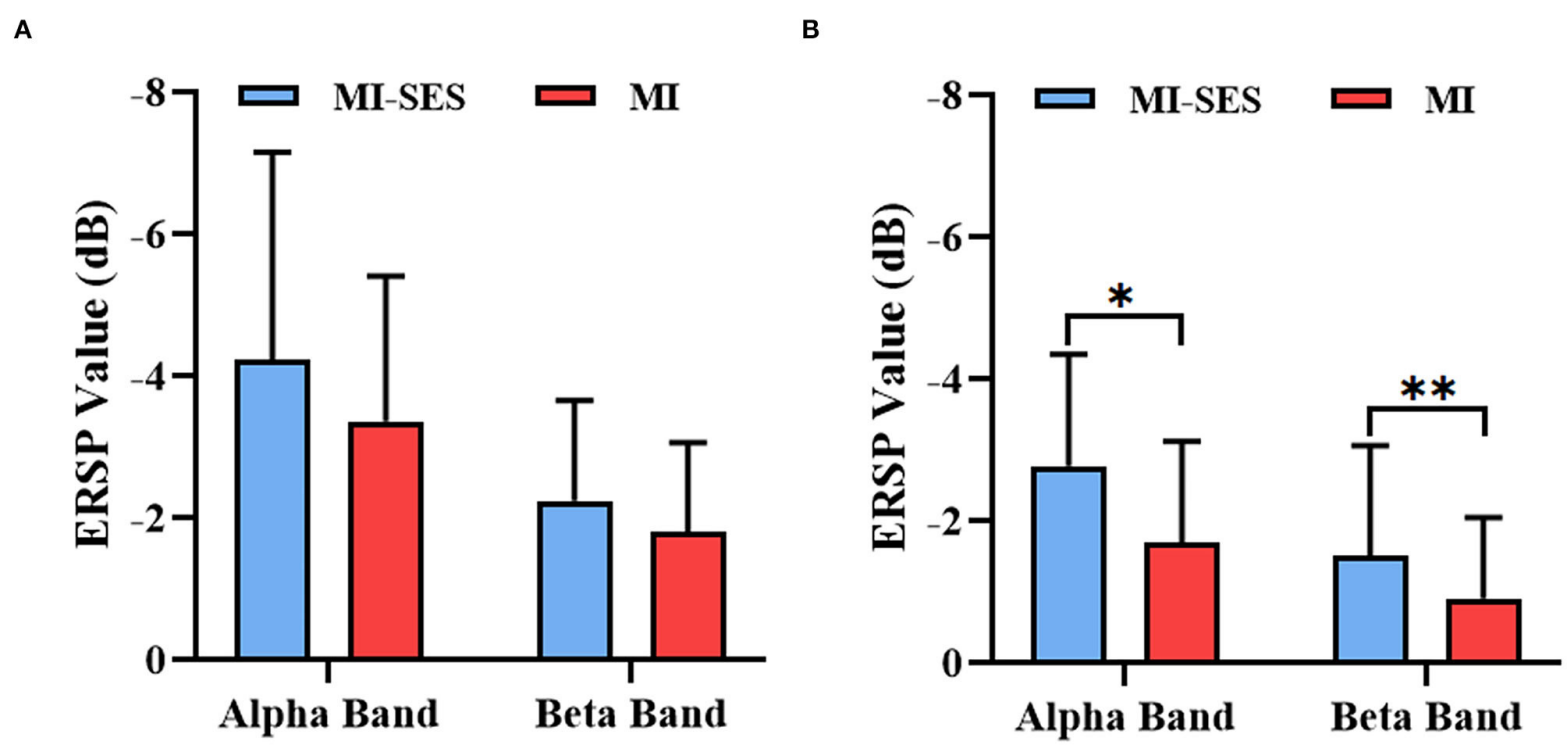
Averaged ERSP values of alpha (8–13 Hz) and beta (14–28 Hz) rhythms during period [3 5] s for MI-SES and MI conditions. **(A)** High Performers group. **(B)** Low Performers group. Error bars represent the standard deviation. “*” indicates *p* < 0.05, “**” indicates *p* < 0.01.

Additionally, in order to quantitatively analyze the effectiveness of st-SES on sensorimotor cortical activation, we compared averaged ERSP values between MI-SES and MI conditions in different EEG frequency bands, including the alpha (8–13 Hz) and beta (14–28 Hz) (Figure 5). The Shapiro–Wilk test indicated that the averaged ERSP values in the alpha band for both High and Low Performers group were normally distributed (*p* > 0.05). In the beta band, the averaged ERSP values in the MI condition of High Performers group (*p* = 0.034) and the MI-SES condition of Low Performers group (*p* < 0.01) were not normally distributed, as assessed by Shapiro–Wilk test. For High Performers group, the averaged ERSP values of both alpha and beta bands were lower in MI-SES condition compared with the MI condition, yet these differences were not significant (alpha: paired *t*-test, *p* = 0.065; beta: Wilcoxon signed-rank test, *p* = 0.139). In contrast, for the Low Performers group, the averaged ERSP values of both alpha and beta bands in MI-SES condition were significantly smaller than those under MI condition (alpha: paired *t*-test, *p* = 0.018; beta: Wilcoxon signed-rank test, *p* < 0.01). The unpaired *t*-test confirmed that there was no significant difference in average ERSP values in the SES condition between the High Performers group and Low Performers group (alpha: *p* = 0.379; beta: *p* = 0.11). These results suggested that somatosensory stimulation combined with motor imagery could significantly promote lateralized sensorimotor cortex activities in Low Performers.

**Figure 6:**
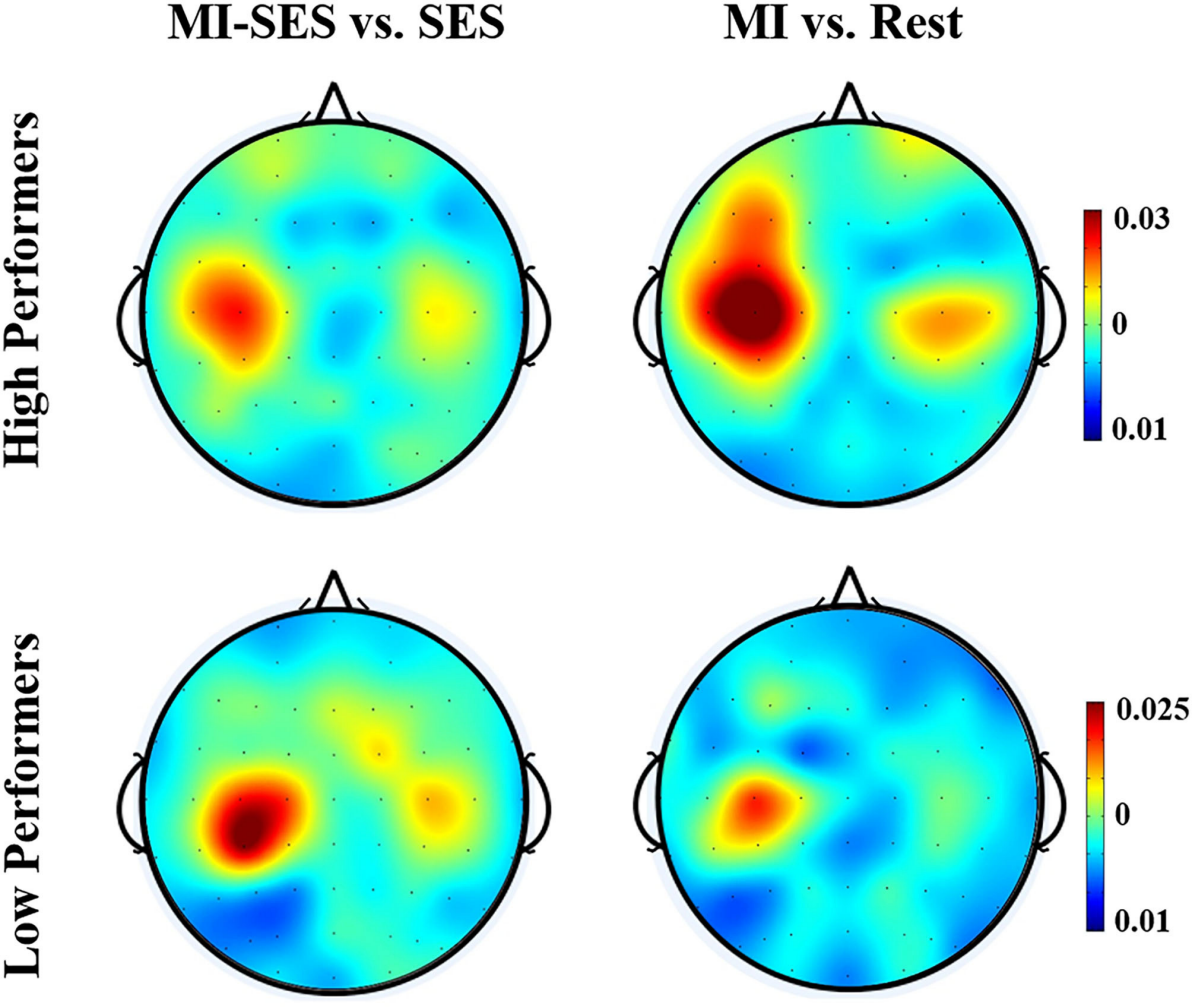
Averaged topographical distributions of *r*^2^ during period [3 5] s for all High Performers and all Low Performers.

The spatial distributions of *r*^2^ values for the High Performers and Low Performers groups are presented in Figure 6. The topographies of the *r*^2^ values revealed that the discriminative information was mostly focused on the left somatosensory cortex. For High Performers group, the *r*^2^ values for comparison between MI-SES and SES conditions were lower than those for comparison between MI and Rest conditions, implying that st-SES modulation reduced the separability of neural patterns between MI and resting state. On the contrary, for the Low Performers group, st-SES modulation elicited an enhancement in *r*^2^ values for comparison between MI and resting state.

### Sample Entropy Results

**Figure 7:**
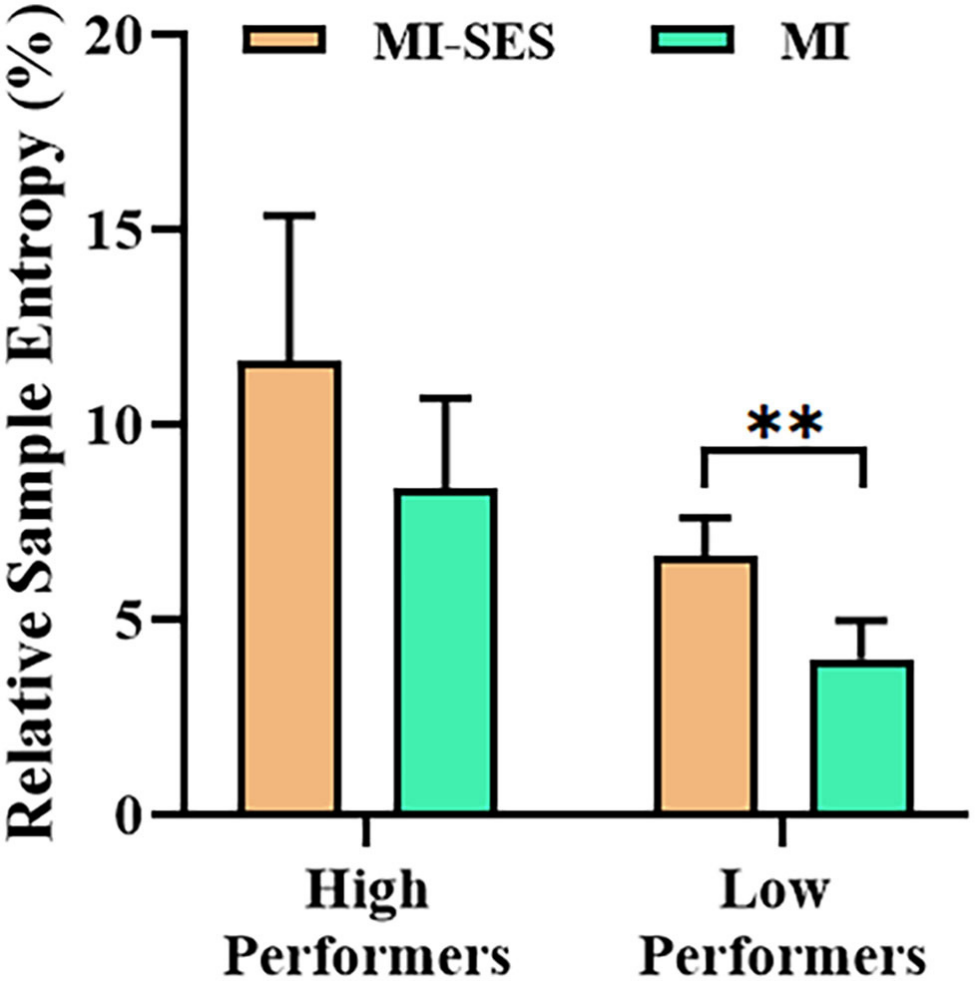
Averaged relative sample entropy values of C3 channel in MI-SES and MI conditions. Error bars represent standard error of mean. “**” indicates *p* < 0.01.

The SampEn values provided further insight into the effects of somatosensory stimulation on EEG complexity. The relative SampEn values of channel C3 in MI-SES and MI conditions for both High Performers and Low Performers are presented in Figure 7. Both MI-SES and MI conditions showed higher relative SampEn values in High Performers group compared with Low Performers group. The relative SampEn values in MI-SES condition for High Performers were not normally distributed, as assessed by Shapiro–Wilk test (*p* = 0.032). In the High Performers group, although the relative SampEn values under MI-SES conditions were greater than those in the MI condition, there was no significant difference in the relative SampEn values of the two conditions (Wilcoxon signed-rank test, *p* = 0.169). For the Low Performers group, however, we found that the relative SampEn values were significantly higher in the MI-SES condition compared with the MI condition (paired *t*-test, *p* < 0.01).

### Functional Connectivity Results

**Figure 8:**
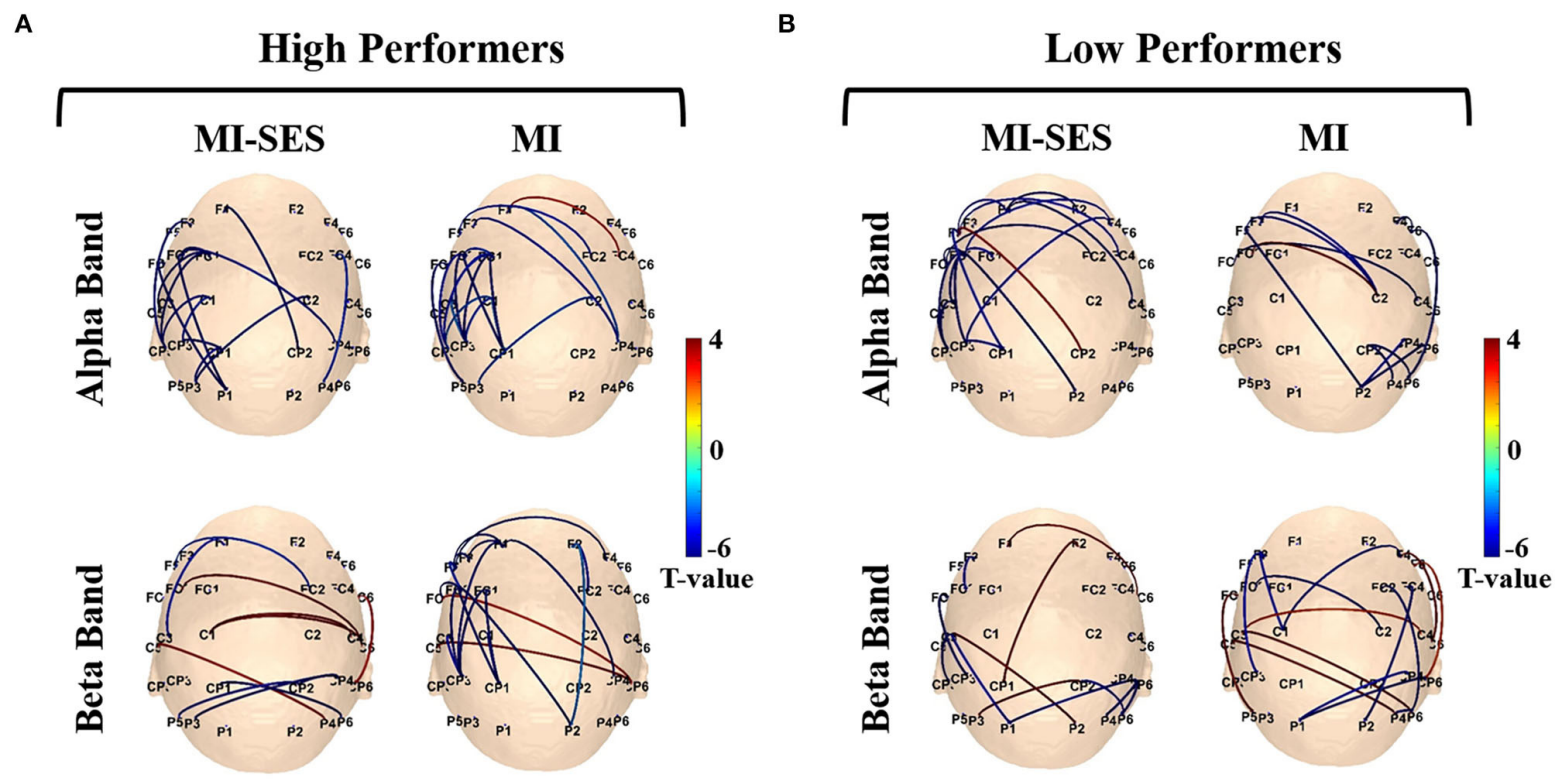
The connectivity networks of alpha and beta rhythms for MI-SES and MI conditions. **(A)** High Performers group. **(B)** Low Performers group. The line between channels represents functional connectivity with significantly different wPLI values between MI and resting states.

Figure 8 shows the functional connectivity alterations between MI task and resting state in each group. These alterations in functional connectivity involved both intra-hemispheric and inter-hemispheric interactions. Notably, we detected phase consistency of the contralateral sensorimotor network decreased significantly in the MI task compared with the resting state. In the High Performers group, a lateralized network at the alpha band could be clearly observed in the contralateral hemisphere during MI-SES and MI conditions. However, no obvious differences in the frontoparietal network were found between the MI-SES condition and MI condition in the alpha band. In contrast, for the beta band, the number of functional connectivity in the left fronto-parietal network was higher in the MI condition compared with the MI-SES condition. For the Low Performers group, a lateralized network at the alpha band was only found in the MI-SES condition, but not in the MI condition. In the beta band, there were no obvious differences in the contralateral fronto-parietal network between the MI-SES condition and the MI condition.

## Discussion

In the present study, we investigated the effect of the combination of st-SES and MI on subjects with different BCI performances. The results demonstrated that st-SES combined with MI fostered the decoding accuracy of brain-switch BCI in Low Performers group, but resulted in a decrease in the brain-switch BCI accuracy in High Performers group. In addition, we found that st-SES only improved the neural response patterns during MI in Low Performers group, but not in High Performers group.

### Differences in Classification Performance

MI-BCI is an effective tool to promote recovery in stroke patients. However, most users are unable to generate reliable neural response patterns to control MI-BCI effectively. This limits the application of MI-BCI in clinical practice. Over the past few decades, intensive research have been conducted to improve the performance of MI-BCI. Some researchers are devoted to increasing MI-BCI accuracy by developing feature extraction and classification algorithms (Chen et al., 2020; Hang et al., 2020). Although these techniques have achieved some success, further improvements in BCI performance are still impeded by the inconsistent MI patterns produced by subjects unfamiliar with MI (Vidaurre et al., 2019; Ren et al., 2020). Thus, it is necessary to develop suitable MI guidance strategies to help subjects understand how to perform MI properly.

Visual and somatosensory stimulation are two commonly used guidance strategies (Ren et al., 2020). Compared with visual stimulation, somatosensory stimulation has the advantage of not occupying the vision and providing more natural guidance for MI tasks (Zhang et al., 2021). St-SES, which can provide somatosensory afference to the targeted limb, has been demonstrated to enhance the activation of the sensorimotor cortex during MI tasks and the performance of MI-BCI (Corbet et al., 2018). However, the results in this study show that the guidance strategy based on st-SES does not seem to be effective in improving the MI-BCI accuracy for all subjects. With the integration of st-SES, the classification accuracy of distinguishing MI task from resting state was significantly improved in the Low Performers group. In contrast, we found that the performance of brain-switch BCI was significantly reduced when st-SES was applied in the High Performance group. Therefore, st-SES may have different effects on neural response patterns in High and Low Performers groups. This interpretation is consistent with the results showing that the *r*^2^ values between MI and resting state were greater during the st-SES intervention in the Low Performers group, where st-SES caused a decrease in the *r*^2^ values for the High Performers group. This indicated that the st-SES intervention made the brain patterns between MI and resting state more separable in the Low Performers group while reducing the distinguishability of neural patterns between MI and resting state in the High Performers group.

In addition, we also observed that the decoding accuracy of the MI-SES vs. Rest classifier was significantly higher than that of the MI vs. Rest classifier only in the Low Performers group, but not in High Performers group. This may be due to the lack of significant difference in sensorimotor cortical activation between MI-SES condition and MI condition in the High Performers group, whereas st-SES induced greater cortical activation during MI in the Low Performers group. This interpretation is consistent with the results showing that the significant differences in ERSP values between MI-SES and MI conditions were found only in the Low Performers group, but not in the High Performers group. However, these findings are inconsistent with a previous study in which the classification accuracy was significantly greater with MI-SES condition compared with MI condition. In fact, Corbet et al. found that st-SES modulation contributed to the improvement of the MI-BCI performance when the data of the first day was used as the training set and the data of the second day was used as the test set (Corbet et al., 2018). When the same day data was divided into training and test sets, there was no significant difference in classification performance between the MI-SES condition and the MI condition. Vidaurre et al. showed that whereas decoding neural patterns from MI conditions using a classifier trained on data from MI-SES conditions results in better classification accuracy, st-SES had no significant effect on the accuracy of identifying MI tasks in MI-SES conditions (Vidaurre et al., 2019). In addition, previous studies have not compared the differences in MI-BCI performance between the High and Low Performers groups during the st-SES intervention (Corbet et al., 2018; Vidaurre et al., 2019). This may obscure the reality that st-SES has different effects on different categories of subjects.

### Dissimilarity in Neural Response Patterns

It has been previously demonstrated that somatosensory stimulation combined with MI can increase sensorimotor cortical activation intensity. The results of this study further indicated that st-SES had different effects on neural patterns of MI in high and low BCI performers. As demonstrated in time-frequency analysis, the sensorimotor cortex activation in Low Performers group, evaluated with averaged ERSP values, was significantly higher during the MI-SES condition than the MI condition. In the High Performers group, however, no significant difference in averaged ERSP values was found between the MI-SES and MI conditions, neither in the alpha or beta bands. One possible interpretation for these results is that differences in motor memory of a given movement resulted in varying feelings of st-SES intervention between the High Performers and the Low Performers. MI has been defined as a mental event in which motor memory of a prior movement is retrieved from the brain, resulting in the experience of re-performing the movement (Lacourse et al., 2005). Indeed, experts with better motor memory performance produce more effective motor imagery for specific movements than novices (Fourkas et al., 2008; Wei and Luo, 2010). This is consistent with our finding that the amplitude of ERD patterns of MI task was higher in the High Performers group compared with the Low Performers group. However, MI involves not only the mental simulation of a movement but also the anticipation of sensory consequences of imagined movements (Kilteni et al., 2018; Vidaurre et al., 2020). Recent evidence has shown that after the motor command is sent, motor imagery, similar to motor execution, predicts both the body state following the upcoming movement and the likely sensory consequences produced by the movement (Kilteni et al., 2018). During motor execution, these predictions are combined with the actual sensory feedback of movement to generate an accurate estimate of the body state. For motor imagery, the representation of our body state can be built up by somatosensory afferents from external devices (Corbet et al., 2018). Moreover, the artificial somatosensory afference, which is more consistent with sensory feedback generated by actual action, is more beneficial for properly representing the body state (Tajadura-Jiménez et al., 2017). Appropriate body representation is critical for MI performance (Ehrsson et al., 2003). Although the st-SES intervention could provide proprioceptive information, there was still a gap between this information and the sensory feedback elicited by the actual movement. High Performers with more efficient motor memory may produce more accurate sensory predictions for a given imagined movement. As demonstrated by Mihara et al., sensory feedback inconsistent with the neural responses failed to induce enhancement in cortical activation (Mihara et al., 2012). The High Performers may be more likely to perceive mismatches between the somatosensory afferents elicited by st-SES and sensory predictions. This may be detrimental to the formation of better body representations through st-SES in the High Performers, which resulted in the sensorimotor cortical activation under MI-SES condition was not significantly different from that in the MI condition. In contrast, Low Performers may be unable to produce accurate sensory predictions according to motor memory during MI. The previous study has shown that new memories associated with external interventions are created when existing memories cannot predict sensory consequences accurately and precisely (Oh and Schweighofer, 2019). Thus, st-SES intervention may help the Low Performers to develop motor memory strategies related to artificial somatosensory afference during MI. This interpretations is consistent with the results showing that the ERSP values of MI-SES condition were significantly smaller than those of MI condition in the Low Performers group.

Additionally, the results of the analysis in the time-frequency domain showed that there was a clear ERD pattern appeared in the contralateral sensorimotor cortex during the SES condition. This may be the main reason for the poor separability of the signal spectrum between MI-SES condition and SES condition in the High Performers group. The ERD pattern in the SES condition may be due to the somatosensory stimulation elicited by st-SES that focused the subjects' attention on the limb sensation. Indeed, Yao et al. showed that the somatosensory cortex was activated when subjects shifted their somatosensory attention to a body part (Yao et al., 2016).

The activity of brain rhythms can also be quantitively analyzed by signal complexity. The previous study has demonstrated that entropy-based methods can be used to quantify the complex dynamics of brain activity (Song et al., 2012). Sun et al. have shown that the activation of the motor cortex can be represented by the entropy-based estimate (Sun et al., 2017). Also, Hanslmayr et al. demonstrated that the desynchronization of sensorimotor rhythm is positively related to the complexity of information processed in the brain (Hanslmayr et al., 2012). In this study, the relative sample entropy of the MI-SES condition was significantly higher than that of the MI condition in the Low Performers group. In the High Performers group, there was no significant difference in relative sample entropy between the MI-SES condition and the MI condition. This could be attributed to the fact that st-SES intervention promoted the activation of the sensorimotor cortex during MI in the Low Performers group, but had no significant effect on the cortical activation in the High Performers group.

### Variation in Functional Connectivity

Functional connectivity quantifies the information exchange across electrodes, allowing us to inspect the communication between brain regions during the MI task. Previous study demonstrated that connections between motor areas increased during MI, while information exchange was suppressed in the resting state (Li et al., 2019). Therefore, we expected that there was a significant increase in frontal-parietal connectivity during the MI task compared with the resting state. However, the results of wPLI showed that in the contralateral sensorimotor network, the functional connectivity during the MI task was lower than that during the resting state. One possible reason for these results is that the decrease in functional connectivity may be related to the cognitive load required for MI. Indeed, Leeuwis et al. found that functional connectivity during the resting state was greater than that during MI in both BCI illiterate and BCI literate subjects (Leeuwis et al., 2021). Mylonas et al. have shown that the decrements of phase synchronization at the alpha and beta bands are associated with the sensorimotor integration that contributes to motor performance (Mylonas et al., 2016). Also, Tzvi et al. reported that the decrease of alpha phase coupling in frontal-parietal network may reflect the allocation of cognitive resources to promote memory encoding (Tzvi et al., 2018). Therefore, our findings suggest that the decrements in phase synchronization of the sensorimotor network are critical for performing MI tasks. In the Low Performers group, the number of functional connectivity representing alpha phase desynchronization was obviously increased in the MI-SES condition relative to the MI condition and focused on the contralateral sensorimotor cortex. These alterations in functional connectivity may imply that Low Performers performed better MI and had greater sensorimotor cortex activation in the MI-SES condition than in the MI condition. In the High Performers group, there was no discernible difference in the functional connectivity between the MI-SES condition and MI condition in the alpha band. In addition, the number of functional connectivity representing beta phase desynchronization was higher in the MI condition compared with the MI-SES condition. This may imply that the st-SES intervention was not beneficial for the High Performers to perform the MI task. In addition, for the High Performers group, we found that the differences in functional connectivity alterations between the MI-SES condition and the MI condition were mainly observed in the beta band. On the other hand, for the Low Performers group, the differences between the MI-SES and MI conditions focused on the alpha band. This may be due to the distinct roles of the alpha and beta bands during MI. Previous studies demonstrated that alpha rhythm is associated with memory retrieval, whereas beta rhythm is involved in motor planning and motor preparation (Tzagarakis et al., 2010; Meirovitch et al., 2015). As mentioned above, st-SES intervention may have a positive effect on motor memory in the Low Performers group, but interfere with the process involving sensory prediction in the High Performers group.

### Limitations of Current Study

Some limitations of the current study need to be mentioned. First, although the different effects of the st-SES intervention on high and low BCI performers have been validated in this study, more subjects should be recruited to draw a generalized conclusion. In particular, we found that some subjects in Low Performers group did not achieve better brain-switch BCI classification performance with st-SES intervention. Although this study has underlined the fact that st-SES may be promising to improve neural responses and MI-BCI performance in the low BCI performers, further studies on greater sample size are needed to clarify the effect of the st-SES intervention. Second, the sensations induced by st-SES may vary depending on the frequency and intensity of the stimulation. Thus, further studies need to be carried out to clarify the effect of different stimulation parameters on the subjects. Third, the effects of somatosensory afference investigated in this study were specific to st-SES. The influence of guidance based on other types of somatosensory afference, such as vibrotactile stimulation, needs to be investigated in the future.

In addition, for the majority of subjects in the Low Performers group, the MI-SES vs. SES classifier achieved better classification performance than MI vs. Rest classifier even in the presence of ERD pattern induced by st-SES intervention in the SES condition. Therefore, the somatosensory input based on st-SES may contribute to the BCI system to better distinguish subjects' neural states between active control and attentive idleness. This is beneficial for low BCI performers to achieve asynchronous control of the BCI system. Further studies involving the application of st-SES intervention in the online BCI system will be needed to understand the advantages and limitations of the st-SES intervention in asynchronous control.

## Conclusions

The main purpose of our work consists in exploring the effect of st-SES on motor imagery tasks in high and low BCI performers. Our findings showed that st-SES intervention improved brain-switch BCI performance in the Low Performers group, but led to a decrease in BCI performance for the High Performers group. Moreover, in the Low Performers group, the electrophysiological analysis demonstrated that st-SES combined with MI achieved significantly higher sensorimotor cortical activation than MI alone. For the High Performers group, st-SES had no significant effect on neural responses during MI. We also found significantly decreased functional connectivity in the frontal-parietal network during the MI task compared with the resting state, and this decrease in functional connectivity may contribute to the execution of the MI task. Notably, st-SES affected the functional connectivity alterations at the beta band in the High Performers group and the alpha band in the Low Performers group, respectively. These results altogether indicated that st-SES intervention may be promising to improve the MI-BCI performance and sensorimotor cortical activation during MI in the Low Performers group but not in the High Performers group.

## Data Availability Statement

The original contributions presented in the study are included in the article/supplementary files, further inquiries can be directed to the corresponding author/s.

## Ethics Statement

This study was reviewed and approved by the Ethics Committee of Tianjin University. The patients/participants provided their written informed consent to participate in this study.

## Author Contributions

LC, LZ, and DM designed the research. LZ conducted the experiment and wrote the original draft. LZ and ZW analyzed the data. BG visualized the results. LC and XZ reviewed and edited the manuscript. All authors contributed to the article and approved the submitted version.

## Funding

This work was supported by the National Natural Science Foundation of China (Nos. 81925020, 82001939, 62006171, and 82102174) and the Tianjin Key Technology R&D Program (No. 20JCYBJC00930).

## Conflict of Interest

The authors declare that the research was conducted in the absence of any commercial or financial relationships that could be construed as a potential conflict of interest.

## Publisher's Note

All claims expressed in this article are solely those of the authors and do not necessarily represent those of their affiliated organizations, or those of the publisher, the editors and the reviewers. Any product that may be evaluated in this article, or claim that may be made by its manufacturer, is not guaranteed or endorsed by the publisher.
